# Neuroanatomical and functional correlates in borderline personality disorder: A narrative review

**DOI:** 10.1002/ibra.12190

**Published:** 2024-12-20

**Authors:** Giulio Perrotta

**Affiliations:** ^1^ Psychology and Psychotherapy Istituto per lo Studio delle Psicoterapie (I.S.P.) Rome Lazio Italy

**Keywords:** amygdala, borderline, borderline personality disorder, limbic system, prefrontal

## Abstract

Borderline personality disorder (BPD) is a dysfunctional, stable, and pervasive alteration in personality functioning with the inability to adapt to the environment, mental rigidity, and ego‐syntonic. High suicidality in BPD patients underlines the significance of research into its pathology. While extensive research on the psychological and behavioral manifestations of BPD can be found in literature, the neuropsychological aspects of the disorder are still partially unknown, although the roles of certain brain structures in the manifestation of the pathology, such as the amygdala, hippocampus, insula, medial prefrontal and cingulate cortices, nucleus accumbens, and temporo‐occipital areas, have already been clarified. This review aims to synthesize current knowledge of the neuroanatomical and functional correlates of BPD, providing insights that may inform future research and therapeutic strategies.

## Graphical abstract

Borderline personality disorder (BPD) is considered a dysfunctional, stable, and pervasive alteration in personality functioning with the inability to adapt to the environment, mental rigidity, and ego‐syntonic, and like all personality disorders is a consistent pattern of inner experience and behavior that deviates markedly from the expectations of the individual's culture, is pervasive and inflexible, onset in adolescence or early adulthood, is stable over time and results in distress or impairment.

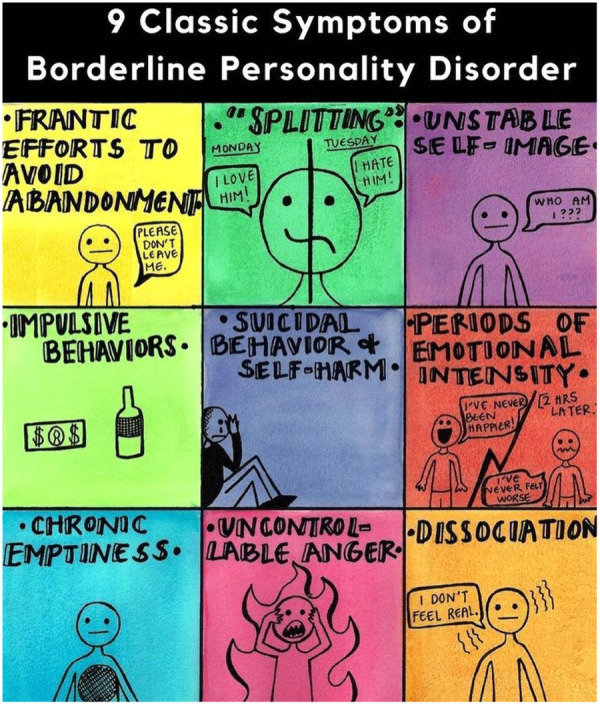

## 1 INTRODUCTION

Borderline personality disorder (BPD) is considered a dysfunctional, stable, and pervasive alteration in personality functioning with the inability to adapt to the environment, mental rigidity, and ego‐syntonic. Like all personality disorders, as a consistent pattern of inner experience and behavior that deviates markedly from the expectations of the individual's culture, it is pervasive and inflexible, typically onset in adolescence or early adulthood, which is stable over time and results in distress or impairment. This disorder is configured in cluster B of personality disorders, along with narcissistic, histrionic, and antisocial disorders, according to the Diagnostic and Statistical Manual of Mental Disorders (DSM‐5‐TR) classification.^1^, ^2^, ^3^, ^4^ Same nosographic placement is in the Perrotta Integrative Clinical Interviews (PICI‐3), which, however, also integrates depressive personality disorder, bipolar personality disorder, and psychopathic personality disorder.^5^ While considerable research has been conducted on BPD, the majority of studies have primarily focused on psychological aspects. As a result, there is a relative paucity of research on its neuropathology. Therefore, this article aims to provide a comprehensive summary of BPD from the perspective of neuropathology.

**Figure 1:**
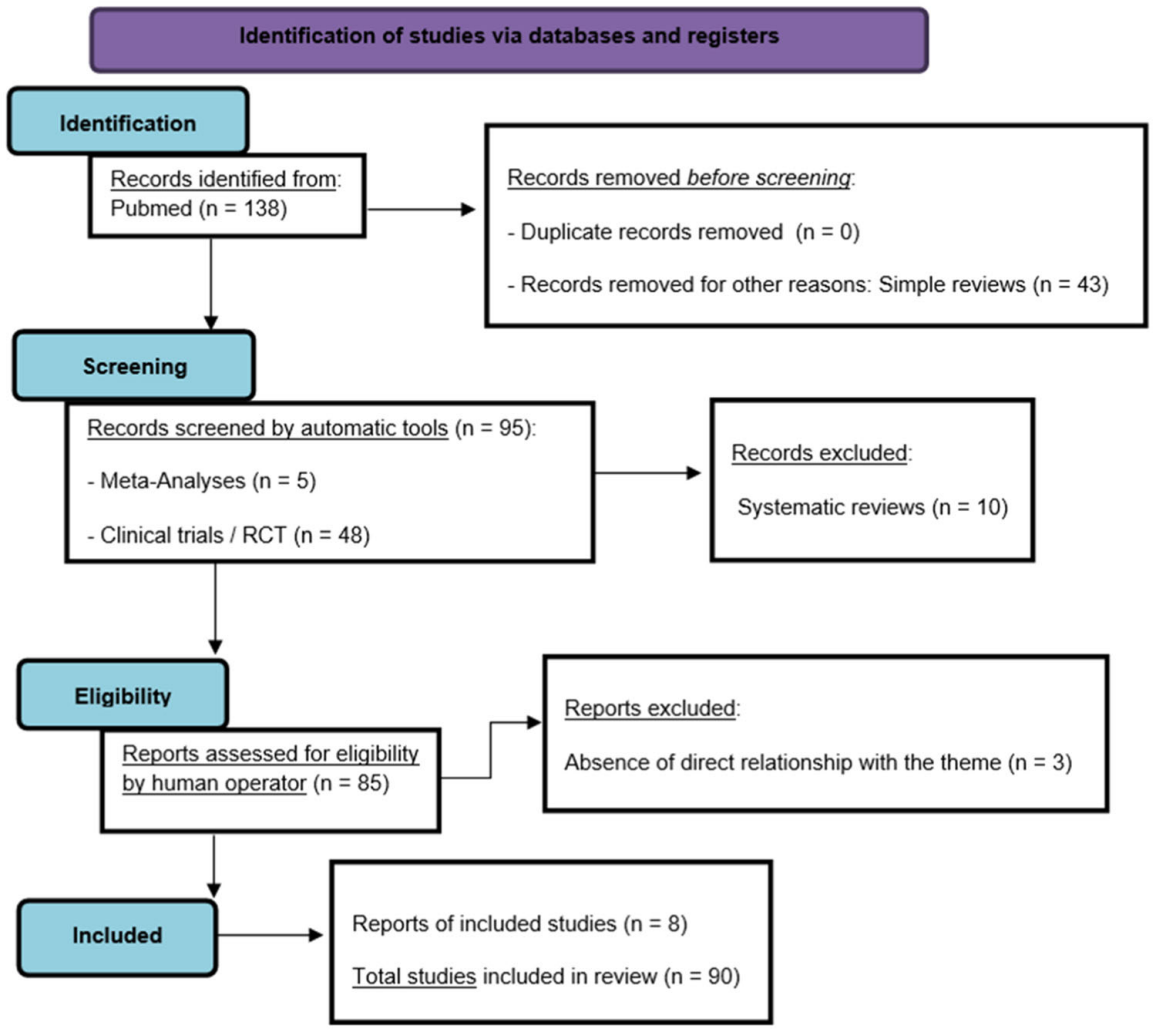
The preferred reporting items for systematic reviews and meta‐analyses (PRISMA) flow diagram template.^6^ [Color figure can be viewed at wileyonlinelibrary.com]

The author searched PubMed, from January 2000 to January 2024, for meta‐analyses, clinical trials, and randomized controlled trials, using “BPD” and “neural correlates,” selecting 138 eligibility results, of which 82 were included by removing duplicate content, irrelevant items, and absence of search items. Then, to get an even broader and more complete overview of the topic, 8 more references were added, for a total of 90 results. Simple and systematic reviews, opinion contributions, or publications in popular volumes were excluded because they were not relevant or redundant for this work. The search was not limited to English‐language papers (Figure 1).

## 2 DESCRIPTIVE, CLINICAL, AND DIAGNOSTIC ELEMENTS OF BPD

According to the DSM‐5‐TR,^1^ BPD is characterized by a pervasive pattern of instability referring, so‐principally, to interpersonal relationships, self‐image, and affect; all of which are characterized by marked impulsivity and emotional dysregulation. The symptomatic picture begins within early adulthood and is present in a variety of contexts. The disorder is associated with significant and widespread impairment in the life of the person with the disorder. The most up‐to‐date incidence figure puts BPD at around 6% of the population, but there are types of populations where the percentage is significantly higher (even up to 30%).^7^, ^8^, ^9^, ^10^, ^11^, ^12^, ^13^, ^14^ Here are the diagnostic criteria that there must be at least five to confirm a diagnosis of BPD:

1. Desperate efforts to avoid real or imagined abandonment.
2. A pattern of unstable and intense relationships, characterized by alternating between extremes of hyper‐idealization and devaluation.
3. Identity alteration: markedly and persistently in‐stable self‐image or perception.
4. Impulsivity in at least two areas that are potentially harmful to the subject (e.g., reckless spending, sex, substance abuse, reckless driving, and binge drinking).
5. Recurrent suicidal behavior, gestures or threats, or self‐mutilating behavior.
6. Affective instability due to marked mood reactivity (e.g., episodic intense dysphoria, irritability, or anxiety, usually lasting a few hours and only rarely more than a few days).
7. Chronic feelings of emptiness.
8. Inappropriate, intense anger or difficulty controlling anger (e.g., frequent angry outbursts, constant rage, and recurrent physical confrontations).
9. Transient paranoid ideation, associated with stress, or severe dissociative symptoms.

According to PICI‐3,^5^ BPD is a habitual, stable, persistent, and pervasive pattern, with onset around age eight but evolves structurally in adolescence, and adulthood is characterized by emotional instability, sudden mood swings, and impulsivity. Here are the diagnostic criteria that there must be at least five to confirm a diagnosis of BPD:

1. Emotional instability and/or impulsiveness in interpersonal relationships;
2. Sudden mood swings;
3. Active and/or passive manipulative tendency;
4. Desperate efforts to avoid abandonment (real and/or imagined);
5. Dysfunctional and/or unstable self‐image;
6. Marked impulsiveness capable of harming them;
7. Persistent feelings of emptiness;
8. Sudden anger and unwarranted aggression;
9. Irrational thoughts and beliefs lead in whole or in part to the psychotic sphere.

However, in literature before 2021, there was no clear classification on the basis of the severity of the disorders, and therefore proceeded in this sense to try to frame it according to a precise rating scale, starting from its characteristic traits, drawing a psychodiagnostic profile that tends from a simple typical characterization in highly functional elevations to the highest level of dysfunction and pathological impairment; specifically, the diagnosis, as framed in PICI‐3, is based on the level of insight present and how many specific dysfunctional traits emerge in the patient's personological picture, according to the Perrotta Borderline Disorder Gravity Scale: (1) excellent, if it has 5 traits; (2) good, if it has 6 traits; (3) mediocre, if it has 7 traits; (4) low, if it has 8 traits; (5) poor, if it has 9 traits.^7^

BPD is, in psychopathology, represented as a relationship disorder, which then decompensates the patient based on the deep anxieties that determine the activation of the primal trauma of abandonment. Individuals with this disorder experience relationships with the constant fear that they may be abandoned by the people to whom they bond. Sometimes, this fear is not expressed through words but is acted out through aggressive (self‐directed and hetero‐directed), controlling, and devaluing behaviors toward one's partner and has as its outcome precisely the one most feared: rejection and abandonment. They constantly oscillate between idealization and devaluation of the other (and themselves) as if imprisoned in a vortex that constantly changes the direction of rotation, never stopping. The deep‐rooted belief in inadequacy and inferiority causes them to experience anxiety and depressive experiences, even to the point of extreme acts such as self‐harm, substance abuse, and even suicide. Research points out that underlying this disorder is a deficit in social cognition (understood as an individual's ability to recognize intentions and emotions in others and thus adjust their behavior during social interactions). The acquisition of this ability is related to an insecure attachment bond with one's caregiver: the attribution of meaning to one's emotional experiences develops through the mirroring exercised by the attachment figure. According to some scholars, therapeutic intervention with this type of patient should aim to strengthen precisely the capacity for mentalization through a “secure” therapeutic relationship. A child whose emotional experiences are not acknowledged will not be able to correctly discriminate what he or she feels, even holding himself or herself responsible for it. This relational mode with the caregiver will only throw him into the chaos of ideas and feelings, feeding over time the anger that will be poured out on others and himself.^15^, ^16^, ^17^, ^18^, ^19^, ^20^, ^21^, ^22^, ^23^

Cognitively, it is not uncommon to find cognitive distortions, dissociative episodes, and also deficits in executive functions, such as attention, concentration, and memory processes. Some individuals, during periods of high stress, may develop psychotic‐like symptoms of the hallucinatory or paranoid type. Behaviorally, the uncontrolled flurry of emotions can trigger intense arousal states of anxiety, tension, or anger, resulting in impulsive and dysregulated patterns, and self‐injurious and self‐destructive actions. Suicide risk among people with BPD is between 3% and 10%. Most of the problems manifested by individuals with BPD are thus the more or less direct consequence of emotional dysregulation and the attempt to modulate intense emotional reactions; from this perspective, many of the self‐injurious and self‐destructive behaviors typical of the symptom constellation of the disorder can be understood as a product of emotional dysregulation itself. Underlying it would be what Linehan calls “emotional vulnerability,” which in turn consists of three basic elements: very high sensitivity to emotional stimuli, intense reactivity, and a slow return to baseline. Individuals with BPD tend to hyper‐mentalize (over‐attribute intentions and emotions to self and others) in a complex and abstract way.^21^, ^22^, ^24^, ^25^, ^26^

## 3 THE MAIN NEUROANATOMICAL AND FUNCTIONAL CORRELATES OF BPD

Recent technological developments in the field of neuroradiology have helped to study the structures and function of the brains of BPD patients in a more timely manner, documenting the neurobiological abnormalities of chronic.^27^

Biological factors appear to play a major role in the development of the disorder. It has been shown, for example, that people with BPD have lower serotonergic functioning than control groups. Furthermore, several studies suggest the contribution of biological factors in the dimension of emotional regulation, emphasizing in particular the involvement of the limbic system and prefrontal areas in recognizing, processing, expressing, and regulating emotions.^28^, ^29^

In general, although with some differences related mainly to gender, age, the severity of the disorder, and trauma experienced, in adult BPD patients, we see neurobiological alterations, both in structure (volume) and function (neurobiological function), in the amygdala, hippocampus, insula, medial prefrontal cortex, cingulate cortex, nucleus accumbens, and temporo‐occipital areas through the use of fMRIs.^30^, ^31^, ^32^

### 3.1 Amygdala

**Figure 2:**
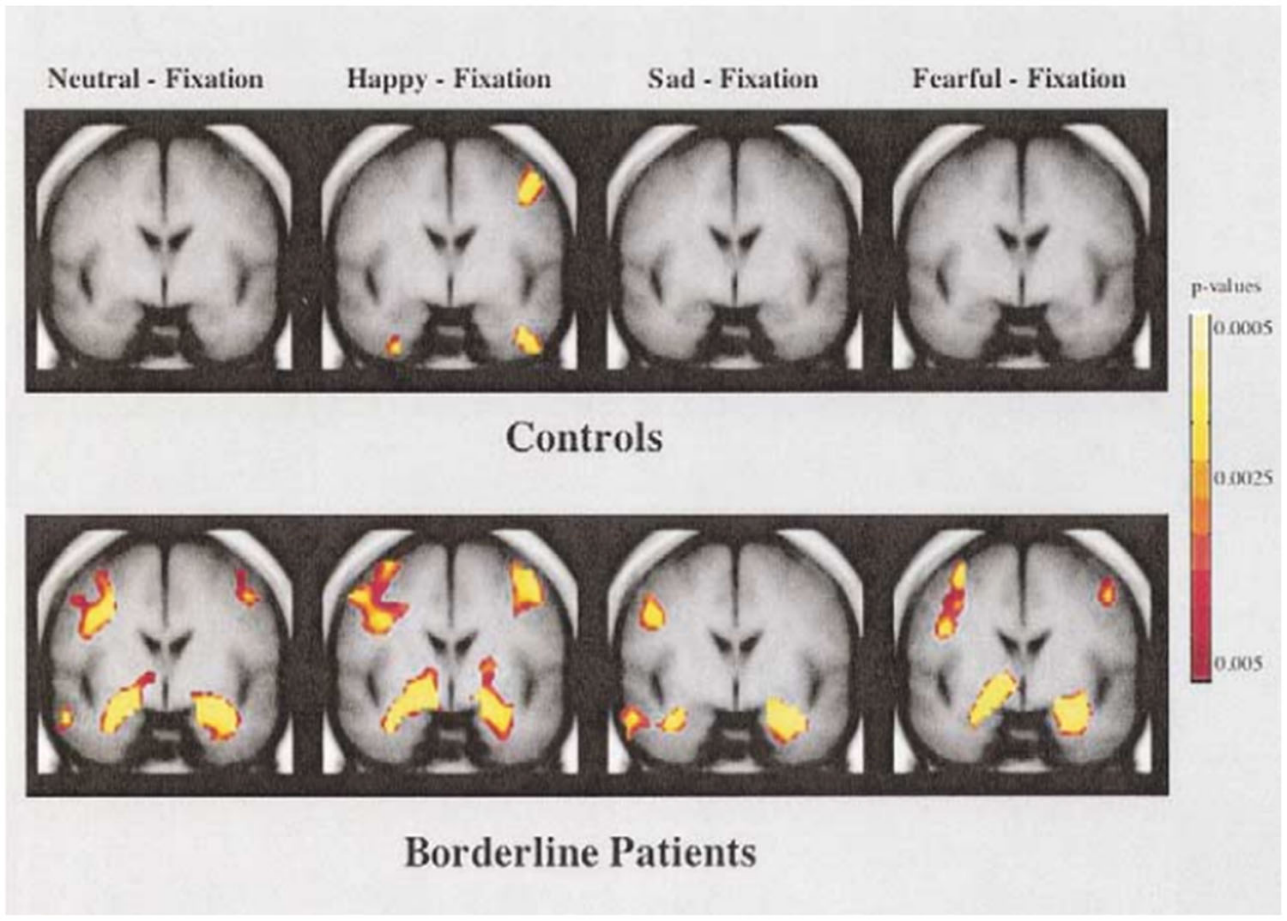
Activation map showing regions in the amygdala slice in which activation exceeded the criterion threshold level of *p* < 0.05 for the normal control and borderline personality disorder groups for each of the four facial expressions.^37^ [Color figure can be viewed at wileyonlinelibrary.com]

The amygdala (or amygdaloid body) is a nuclear complex located in the dorsomedial part of the temporal lobe of the brain that manages emotions. At the anatomical level, it is also defined as a group of interconnected structures of gray matter forming part of the limbic system, located above the brainstem, in the rostromedial region of the temporal lobe, below the uncinate gyrus (uncus) and anterior to the hippocampal formation. It has an ovoid structure (amygdala means almond in ancient Greek) located at the lowest point of the upper wall of the inferior horn of each lateral ventricle and is in continuity with the putamen and as a terminal part with the tail of the caudate nucleus. Under physiological conditions, it is believed to be the center of integration of higher neurological processes such as emotions, but it is also involved in emotional memory systems. It is active in the system of comparing received stimuli with past experiences and in processing olfactory stimuli. Signals from the sense organs first reach the thalamus, then serving a monosynaptic circuit, arrive at the amygdala (there is a very thin bundle of nerve fibers running from the thalamus to the amygdala); a second signal is sent from the thalamus to the neocortex. This branching allows the amygdala to begin responding to stimuli before the neocortex. In this way, the amygdala can analyze every experience by sounding out situations and every perception. When it assesses a stimulus as dangerous, for example, the amygdala snaps like a kind of neural trigger and reacts by sending emergency signals to all major parts of the brain; it stimulates the release of hormones that trigger the attack‐or‐flight reaction, (adrenaline, dopamine, and norepinephrine), mobilizes the movement centers, and activates the cardiovascular system, muscles, and gut. At the same time, mnemonic systems are “flicked through” with absolute priority to recall any useful information in the fear situation. While the hippocampus “remembers” facts, the amygdala judges their emotional valence. The amygdala then gives each stimulus the right level of attention, enriches it with emotion, and finally initiates its storage in the form of memory. The amygdala is thus the repository of our emotional memory, so it analyzes the current experience, with what has already happened in the past: when the present and past situations have a similar key element, the amygdala identifies it as an association and acts, sometimes, before it has full confirmation. It hastily commands us to react to a present situation according to comparisons of similar episodes, even from long ago, with learned thoughts, emotions, and reactions fixed in response to similar events. The amygdala may react before the cortex knows what is happening, and this is because the raw emotion is triggered independently of conscious thought, and generally before it.^33^, ^34^, ^35^ In BPD patients, the amygdala is severely altered in both structural and functional parts. Some studies^36^, ^37^, ^38^ demonstrated bilateral (and especially left) hyperactivation in neutral situations that were, however, interpreted as threatening, including in the context of facial expression recognition, where these subjects tend to interpret as threatening something that is not. Other studies;^39^, ^40^ however, have shown that there can also be episodes of hypoactivation due to prolonged exposure to emotional stimuli that can somehow act as an off switch for the mechanism. In summary, increased activation of the left amygdala and posterior cingulate cortex has been shown, along with alleviated responses at the level of the dorsolateral prefrontal cortex in both cerebral hemispheres during the processing of negative emotional stimuli, while also demonstrating functional hyperactivity and lower gray matter volume at the level of the left amygdala. From a neurobiological perspective,^41^ the threat would be related to increased, but also prolonged, amygdaloid responses, while inadequate emotion regulation would also be attributable to reduced inhibition of the amygdala by prefrontal circuits. Furthermore, it would appear that patients with BPD^42^ have a higher pain threshold, and this could be associated with two mechanisms: the first mechanism involves the deactivation of the amygdala and hyperactivation of prefrontal‐limbic circuits that would reflect cortical inhibitory modulation; the second mechanism involves increased activity in circuits between the posterior insula and the dorsolateral prefrontal cortex that could reflect abnormal pain assessment that contributes to hypoalgesia in BPD. Relative to the structural elements, studies^36^, ^43^ showed a marked tendency for hypovolumetry of the amygdala and the presence of lower gray matter density, especially on the left side. Investigating, finally, possible correlations between age and gray matter alterations, some studies^44^, ^45^ then showed that volumetric abnormalities in prefrontal areas were present from the onset of the disorder (often in adolescence), while gray matter deficits related to limbic areas developed later in the course of the disorder (e.g., in early adulthood), probably showing that as found in some cross‐sectional studies, symptoms of impulsivity (more related to prefrontal dysfunction) would peak during adolescence and then decrease in adulthood, while symptoms of disturbed affectivity and emotionality (more related to limbic system abnormalities) would remain more stable over time (Figure 2).

### 3.2 Hippocampus

**Figure 3:**
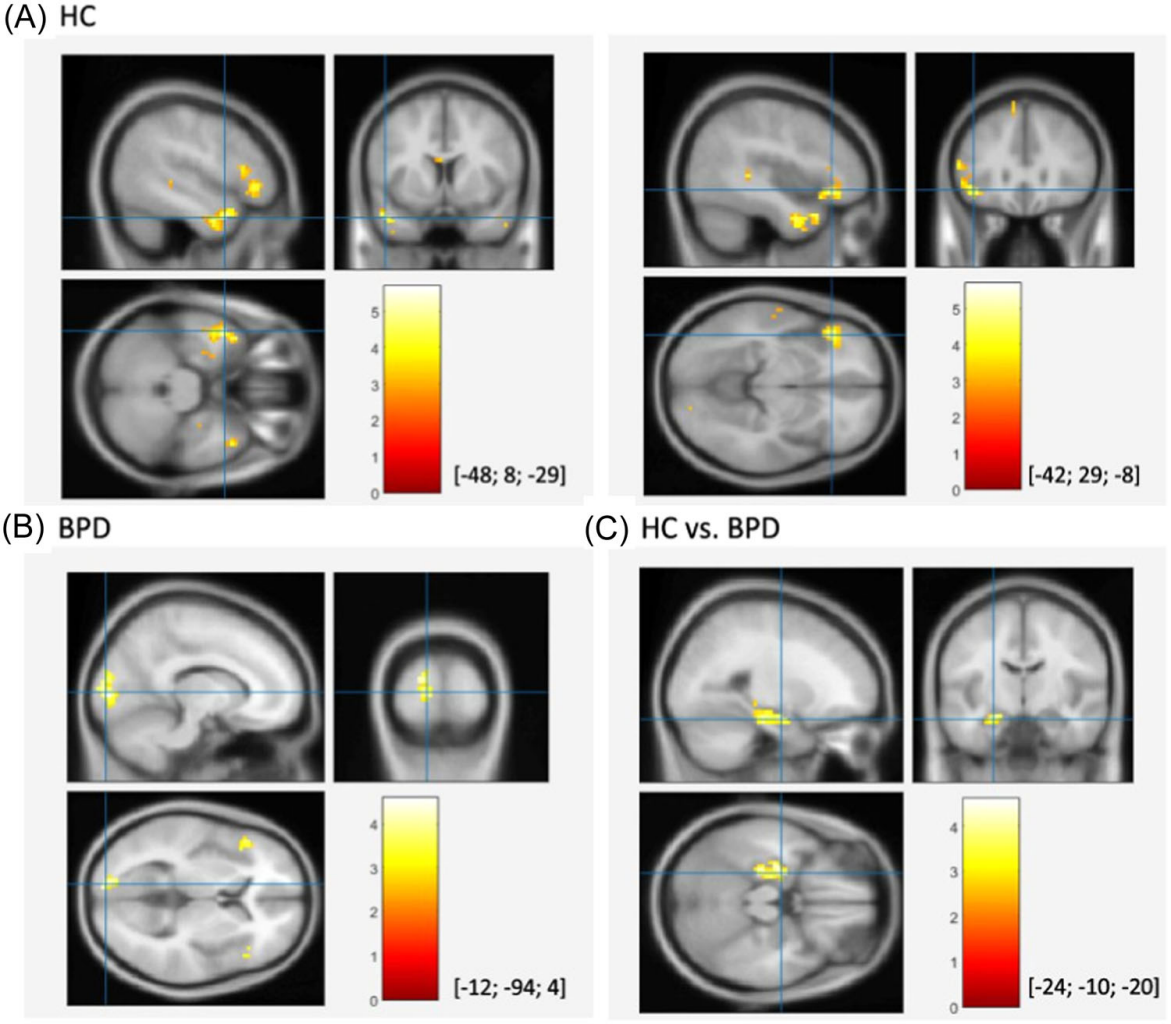
Enhanced neural activation within the left hippocampus during social exclusion compared to social inclusion conditions in the Cyberball paradigm. Results for healthy controls (A), patients with BPD (B), and comparison between the groups (C). Intersection of blue lines indicates the peak of each cluster, MNI‐coordinates are provided in the squared brackets. BPD, borderline personality disorder; HC, healthy control; MNI, Montreal Neurological Institute (*x*, *y*, and *z* coordinates are provided in mm).^56^ [Color figure can be viewed at wileyonlinelibrary.com]

It is located in the inner region of the temporal lobe and is part of the limbic system; it contains two main interlocking parts: the hippocampus proper (also called Ammon's horn) and the dentate gyrus. It plays an important role in the formation of explicit memories (declarative and semantic), in the transformation of short‐term memory into long‐term memory, and spatial navigation.^46^, ^47^, ^48^ Damage to the hippocampus does not affect some aspects of memory, such as the ability to acquire new motor skills (e.g., playing a musical instrument): this suggests that these types of skills depend on a different type of memory (procedural memory) and different brain regions.^49^, ^50^ In BPD patients, there is the presence of a hypo volumetric hippocampus,^36^, ^51^, ^52^, ^53^, ^54^, ^55^, ^56^ sensitive to the use of hydrocortisone, which promotes the recovery of autobiographical memory^57^ and the duration/typology of the trauma.^58^ Reduced hippocampal volume^30^ may be related to the difficulties that BPD patients have in assessing the parallels between current relationships and past ones and in learning from the experiences associated with these previous relationships. Additionally, studies using the Cyberball task, which includes conditions of social inclusion, exclusion, and overinclusion, have shown that BPD patients exhibit heightened neural activation in the left hippocampus during social exclusion compared to social inclusion, further highlighting the role of the hippocampus in the social and emotional processing challenges characteristic of BPD^56^ (Figure 3).

### 3.3 Insula

Insula is a portion of the cerebral cortex that lies deeply within the scissure of Silvius; it can be observed by dividing the two lips of the scissure of Silvius. The cortical area that covers it and separates it from the lateral surface of the brain is the opercula (Latin for “eyelids”). The opercula are formed by parts of the frontal, temporal, and parietal lobes that form a lid for the insula. The insular cortex is divided into two parts: an anterior part (anterior insula), consisting of about three short circumvolutions, and a posterior part (posterior insula), consisting of one or two long circumvolutions. The posterior portion of the insula appears to be smaller, and more than twelve cytoarchitectural fields have been identified there. The insula plays a role in several functions often related to emotionality or the regulation of bodily homeostasis: these functions include perception, motor control, self‐awareness, cognitive functions, and interpersonal experience.^59^, ^60^, ^61^, ^62^ Damage to the structure can lead to the onset of several morbid conditions, such as progressive non‐fluent aphasia and substance addictions, but also anxiety and depression states, and some dementias such as frontotemporal and Alzheimer's disease.^63^, ^64^, ^65^, ^66^, ^67^ In BPD patients, however, there is hyperactivation of the insula area; this structure is critical in the regulation of the autonomic nervous system and the regulation of negative emotions, and its hyperfunctioning is connected with posttraumatic stress symptoms, obsessive disorder, social anxiety, and specific phobia, which in common with BPD have precisely phobic, obsessive, and paranoid symptoms.^53^, ^68^, ^69^, ^70^, ^71^, ^72^

### 3.4 Prefrontal cortex

**Figure 4:**
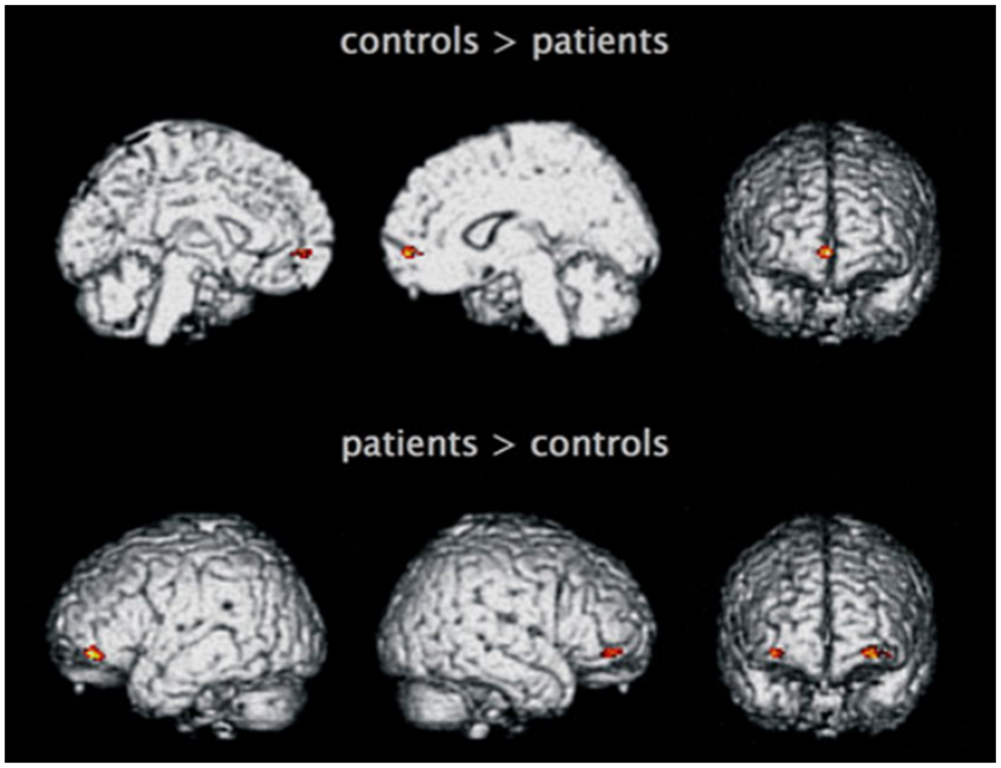
Regions exhibiting decreased and increased regional cerebral blood flow (CBF) in patients with BPD compared to healthy controls.^80^ BPD, borderline personality disorder. [Color figure can be viewed at wileyonlinelibrary.com]

**Figure 5:**
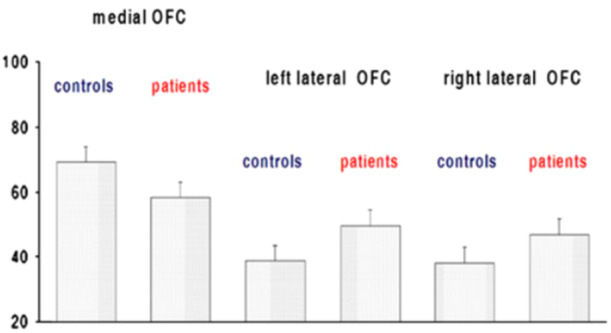
Extracted CBF values (means and standard error) from medial and lateral orbitofrontal cortex (OFC) clusters.^80^ [Color figure can be viewed at wileyonlinelibrary.com]

Prefrontal cortex is the anterior part of the frontal lobe of the brain, located in front of the primary motor cortex and premotor cortex. This area encompasses various Brodmann areas (Brodmann Area 9, Brodmann Area 10, Brodmann Area 11, Brodmann Area 12, and Brodmann Area 46, Brodmann Area 47). The region is involved in planning complex cognitive behaviors, personality expression, decision‐making, and moderation of social conduct. The basic activity of this region is considered to be the guidance of thoughts and actions by one's goals; in fact, extensive structural damage to it leads to impairments in concentration, orientation, abstract skills, sense of judgment, and skill problem solving; destroying the orbital (frontal) lobe, on the other hand, leads to the conduct of inappropriate social conduct. The set of functions assigned to the prefrontal cortex is that, therefore, of the executive system, which is involved in skills such as distinguishing conflicting thoughts, determining good and bad, equal and different, determining the consequences of current activities, working toward a particular goal, predicting outcomes, making expectations based on actions, and social “control,” i.e., the ability to suppress stimuli that might lead to unacceptable social conduct.^73^, ^74^, ^75^ In borderline patients, the prefrontal cortex is severely impaired both structurally and functionally. In particular, the bilateral dorsolateral prefrontal responds abruptly during the processing of negative emotional stimuli, and negatively correlating with the amygdala results in a deficit in emotion regulation mimicking a coordination disturbance between prefrontal and limbic areas (in the narrow sense).^36^ In fact, some evidence has found minor activity (hypoactivity) in areas involved in the cognitive regulation of emotions, such as the dorsolateral prefrontal cortex, medial, orbital, and anterior cingulate cortex.^54^, ^76^, ^77^, ^78^, ^79^, ^80^ Further studies have given findings regarding low gray matter density in the frontal‐dorsal, which may predispose these individuals to memory processing difficulties with particular difficulty and hypersensitivity to memories related to negative experiences and emotions ^81^, ^82^, ^83^ (Figures 4, 5).

### 3.5 Temporal‐occipital areas

**Figure 6:**
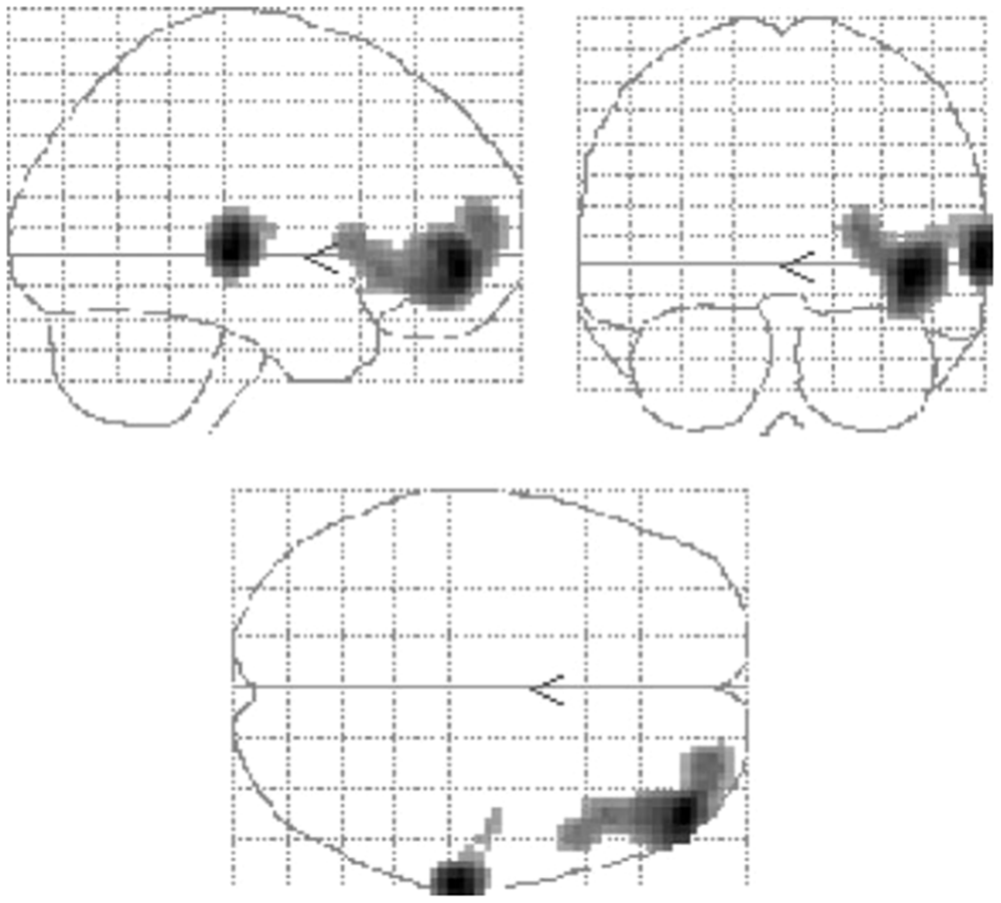
Brain glass images indicating projections of hypoperfusion clusters in impulsivity‐related personality disorder.^85^ [Color figure can be viewed at wileyonlinelibrary.com]

It is the vast area devoted primarily to visual (occipital) and spatial (temporal) perception processes but also to linking with emotional processes and visual and tactile information. In BPD subjects, research shows reduced activation of the superior temporal sulcus and the superior temporal gyrus, as well as also reduced neural activity in two brain regions, the temporoparietal junction and the superior temporal sulcus, which turn out to be of crucial importance during empathic‐type processes. The temporal‐medial cortex also appears to be impaired by low gray matter density and temporofrontal areas (especially on the right) that result in regional cerebral blood flow (rCBF) deficit^46^, ^81^, ^84^, ^85^ (Figure 6).

### 3.6 Orbitofrontal cortex, right parietal lobe and fusiform gyrus

These three areas, which in norm subjects are involved in the cognitive processing and organization of decision making, emotional, physical, and sensory recognition (including facial expressions), and reward processing. In BPD patients, they turn out to have reduced metabolism and low gray matter density, thus promoting misinterpretations of reality, emotional dissipation, and aggressive‐impulsive reactions.^46^, ^81^

### 3.7 Cingulate cortex

Cingulate cortex is divided into three subregions each with a different function: rostral (anterior) involved in emotion; dorsal, involved in cognition; caudal (posterior), involved in motor control. The anterior cingulate circuit, in particular, connects this structure with the orbitofrontal cortex and the amygdala. It forms a pathway from the thalamus to the hippocampus and is also associated with olfactory memory and pain recollection.^34^, ^46^ In BPD individuals, this complex structure has low gray matter density both anteriorly and posteriorly,^81^ hyperactivation in the posterior site (promoting hyperactivity)^36^, ^86^ and hypoactivation in the anterior site (promoting dysfunctionality with the amygdala and the negative perceptual state associated with fear).^87^

### 3.8 Nucleus accumbens

Nucleus accumbens is a region of the basal forebrain, rostral to the preoptic area of the hypothalamus. The nucleus accumbens and the olfactory tubercle collectively form the ventral striatum, which is part of the basal nuclei. It is a structure present on both sides of the telencephalon and is located between the head of the caudate nucleus and the anterior portion of the putamen; it is joined medially to the septum pellucidum. As a whole, the nucleus accumbens plays an important role in the cognitive processes of aversion, motivation, reward, and multiple mechanisms of action reinforcement. It is thought to play an important role in reinforcement mechanisms, laughter, addiction, processing of feelings of pleasure and fear, as well as the onset of the placebo effect. In BPD patients this area is shown to be hypoactive, favoring anxious phenomena due to the probable correlation with dysfunction at the neurotransmitters gamma‐amino butyric acid (GABA) and dopamine.^34^, ^46^, ^88^, ^89^

### 3.9 White matter

White matter is part of the brain and spinal cord that contains nerve fibers. It is often contrasted with gray matter, which instead contains the neuronal cell bodies. The white matter's main job is to transmit information, in the form of electrical impulses, from one part of the brain to another. It thus connects different parts of the brain and cerebellum and is the basis for the importance of the concept of “connectivity.” This happens both between neighboring areas that perform the same function and between very distant areas that must constantly communicate to perform a complex task, such as, for example, recognizing the scent of a rose and calling it by its name. The right hemisphere and left hemisphere, basal nuclei and cerebellum, cerebral cortex, and spinal cord are all interconnected through white matter. Damage to the white matter results in reduced interconnectivity between structures, as occurs in periventricular leukomalacia, single lesions, periventricular hemorrhagic infarction, cytomegalovirus, toxoplasmosis, and rubella infections, and oxidative stress.^34^, ^46^ In BPD patients, research has shown a decrease in the right posterior hemisphere and corpus callosum (correlating with anxious states).^90^

Below is a summary table of the neuroanatomical differences between a subject with BPD and a healthy one (Table 1).

**Table 1:** Neuroanatomical and functional differences between a healthy subject and a subject with BPD.

Neuroanatomical areas	Healthy subject (normal adult)	Person with BPD
*Amygdala*	It is believed to be the center of integration of higher neurological processes such as emotions but is also involved in emotional memory systems. It is active in the system of comparing received stimuli with past experiences and in processing olfactory stimuli	Increased activation of the left amygdala and posterior cingulate cortex was shown, along with alleviated responses at the level of the dorsolateral prefrontal cortex in both cerebral hemispheres during the processing of negative emotional stimuli, also demonstrating functional hyperactivity and lower gray matter volume at the level of the left amygdala
*Hippocampus*	It plays an important role in the formation of explicit memories (declarative and semantic), the transformation of short‐term memory into long‐term memory, and spatial navigation	Hypovolumetric, directly related to the prefrontal cortex and amygdala
*Insula*	It plays a role in several functions often related to emotionality or the regulation of bodily homeostasis: these functions include perception, motor control, self‐awareness, cognitive functions, and interpersonal experience	Hyperactivity
*Prefrontal Cortex*	The basic activity of this region is considered to be the guidance of thoughts and actions by one's goals; in fact, extensive structural damage to it leads to impairments in concentration, orientation, abstract skills, sense of judgment, and skill problem solving	General hypoactivity, with the bilateral dorsolateral prefrontal responding abruptly during the processing of negative emotional stimuli, negatively correlating with the amygdala and resulting in a deficit in emotion regulation. Abnormalities (low) density in cortical gray matter
*Temporal‐occipital areas*	It is the vast area devoted mainly to the processes of visual (occipital) and spatial (temporal) perception but also to the connection with emotional processes and visual and tactile information	Reduced activation of the superior temporal sulcus, superior temporal gyrus, temporoparietal junction, and superior temporal sulcus. The temporal‐medial cortex has low gray matter density, and the temporofrontal areas (especially on the right) have a deficit of cerebral blood flow
*Orbitofrontal cortex, right parietal lobe and fusiform gyrus*	These three areas, which in norm subjects are involved in cognitive processing and organization of decision making, emotional, physical, and sensory recognition (including facial expressions), and reward processing	Reduced metabolism and low gray matter density
*Cingulate cortex*	The anterior cingulate circuit, in particular, connects this structure with the orbitofrontal cortex and the amygdala. It forms a pathway from the thalamus to the hippocampus and is also associated with olfactory memory and pain recollection	Low gray matter density both anteriorly and posteriorly, hyperactivation in the posterior site, and hypoactivation in the anterior site
*Nucleus accumbens*	It plays an important role in the cognitive processes of aversion, motivation, reward, and multiple mechanisms of action reinforcement. It is thought to play an important role in reinforcement mechanisms, laughter, addiction, processing of feelings of pleasure and fear, as well as the onset of the placebo effect	Hypoactivity
*White matter*	Transmission of information, in the form of electrical impulses, from one part of the brain to another	Hypovolumetry in the right posterior hemisphere and corpus callosum

## 4 FUTURE PERSPECTIVE

The topic of neural correlates in BPD patients is complex and multifaceted, and no studies related to correlations between neurobiological aspects and symptom severity can be found in the literature. This point of analysis is crucial to define in a timely manner whether or not there is a statistically significant (increasing or decreasing) relationship. Detecting the possible presence of neural correlates in relation to symptom severity would open the door to a strand of study that can demonstrate with statistical certainty the neurobiological nature of the disorder. The future perspective for research in this area is, therefore, to assess the complexity of the case by taking into account the specific interaction between different variables (biological, psychological, and personological) and to identify a consistent and common profile for the disorder under investigation.

## 5 CONCLUSIONS

BPD is a complex and disabling personality disorder that involves a varied manifestation of symptoms that are reflected in an equal variability of structural and functional brain abnormalities, found mainly in the limbic area, prefrontal cortex, and temporal and parietal lobes. These abnormalities may be related both to possible individual vulnerabilities, which may thus predispose the person to the risk of developing BPD, as well as to consequences of exposure to trauma and reinforcers resulting from the disorder itself, respecting individual differences that depend on sex, gender, severity, duration, and concomitant causes. In conclusion, although knowledge and awareness of BPD and its symptomatological, psychological, and neurobiological features have increased significantly in recent years, leading to the refinement of available therapeutic strategies and the design of new ones, to date an optimal level of effectiveness has not yet been achieved.

## AUTHOR CONTRIBUTIONS

Giulio Perrotta organized, wrote, and revised this article.

## CONFLICT OF INTEREST STATEMENT

The author declares no conflicts of interest.

## ETHICS STATEMENT

Not applicable.

## Data Availability

Not applicable as no new data is generated in this review article.
